# Optogenetic Stimulation of the Corticothalamic Pathway Affects Relay Cells and GABAergic Neurons Differently in the Mouse Visual Thalamus

**DOI:** 10.1371/journal.pone.0045717

**Published:** 2012-09-20

**Authors:** Chris W. D. Jurgens, Karen A. Bell, A. Rory McQuiston, William Guido

**Affiliations:** Department of Anatomy and Neurobiology, Virginia Commonwealth University Medical Center, Richmond, Virginia, United States of America; University College London, United Kingdom

## Abstract

The dorsal lateral geniculate nucleus (dLGN) serves as the primary conduit of retinal information to visual cortex. In addition to retinal input, dLGN receives a large feedback projection from layer VI of visual cortex. Such input modulates thalamic signal transmission in different ways that range from gain control to synchronizing network activity in a stimulus-specific manner. However, the mechanisms underlying such modulation have been difficult to study, in part because of the complex circuitry and diverse cell types this pathway innervates. To address this and overcome some of the technical limitations inherent in studying the corticothalamic (CT) pathway, we adopted a slice preparation in which we were able to stimulate CT terminal arbors in the visual thalamus of the mouse with blue light by using an adeno-associated virus to express the light-gated ion channel, ChIEF, in layer VI neurons. To examine the postsynaptic responses evoked by repetitive CT stimulation, we recorded from identified relay cells in dLGN, as well as GFP expressing GABAergic neurons in the thalamic reticular nucleus (TRN) and intrinsic interneurons of dLGN. Relay neurons exhibited large glutamatergic responses that continued to increase in amplitude with each successive stimulus pulse. While excitatory responses were apparent at postnatal day 10, the strong facilitation noted in adult was not observed until postnatal day 21. GABAergic neurons in TRN exhibited large initial excitatory responses that quickly plateaued during repetitive stimulation, indicating that the degree of facilitation was much larger for relay cells than for TRN neurons. The responses of intrinsic interneurons were smaller and took the form of a slow depolarization. These differences in the pattern of excitation for different thalamic cell types should help provide a framework for understanding how CT feedback alters the activity of visual thalamic circuitry during sensory processing as well as different behavioral or pathophysiological states.

## Introduction

**Figure 1:**
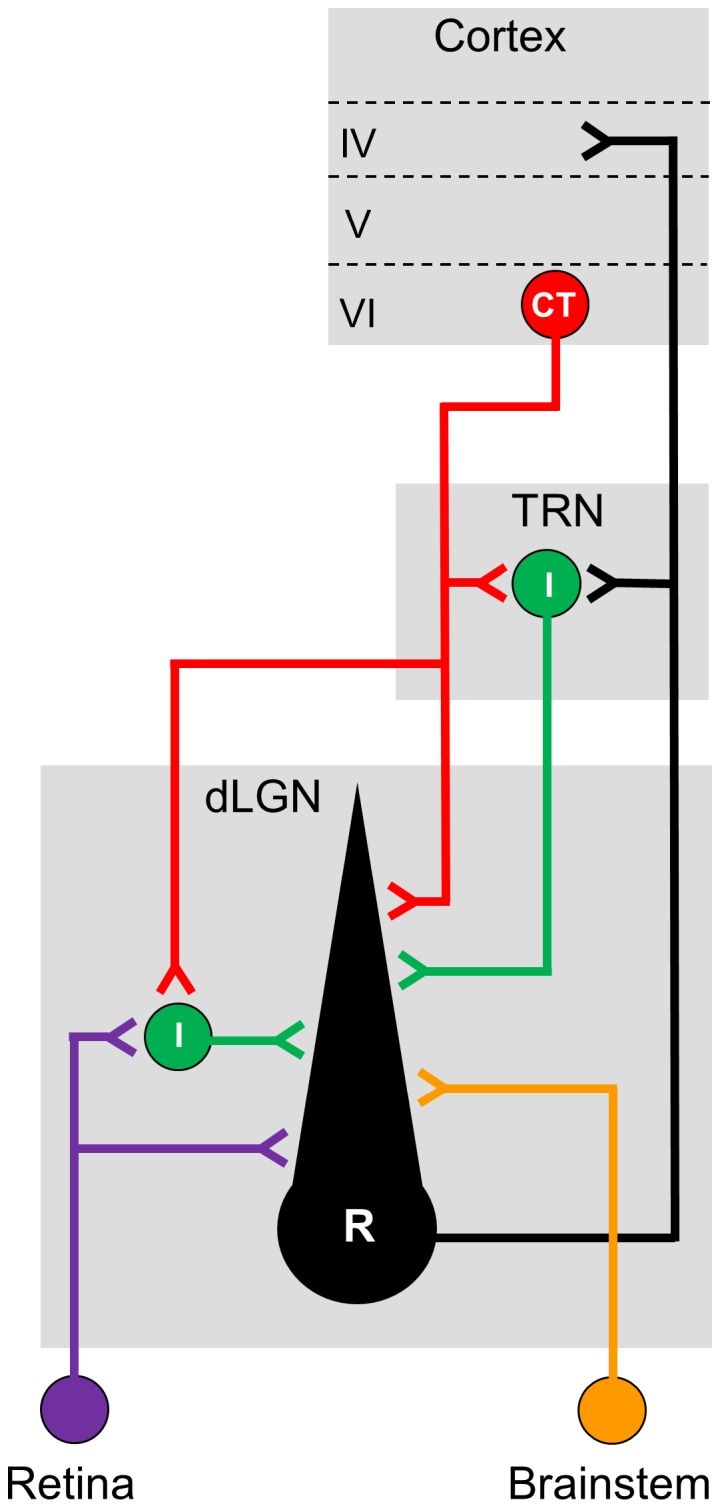
Schematic showing various cell types and circuits associated with dLGN. Relay cells (R, black) of the dorsal lateral geniculate nucleus (dLGN) receive driver-like input from the retina (purple) as well as modulatory input from a variety of nonretinal sources including the brainstem (orange), inhibitory neurons (I, green) of the dLGN and the adjacent thalamic reticular nucleus (TRN), and excitatory neurons of layer VI of the visual cortex (CT, red). Relay cells project primarily to layer IV of visual cortex but also send axon collaterals to GABAergic neurons of TRN. The corticothalamic projections from layer VI neurons innervate relay cells of dLGN as well as GABAergic interneurons of dLGN and TRN. Both sets of thalamic inhibitory neurons form feedforward and feedback connections with dLGN relay cells. For clarity, brainstem projections to inhibitory neurons of dLGN and TRN, as well as the distinction between F1 and F2 inhibitory synapses involving intrinsic interneurons and relay cells are not shown. Grey boxes depict dLGN (bottom), TRN (middle) and cortex (top).

The dorsal lateral geniculate nucleus (dLGN) of the thalamus is the principal relay of retinal information to the visual cortex. However, the vast majority of synapses in this nucleus arise from nonretinal sources and provide a powerful substrate for modulating retinogeniculate signal transmission [1], [2] (see Fig. 1). Perhaps the largest source of nonretinal input to the dLGN is the feedback projection from layer VI cells of the visual cortex [3]–[5]. Corticothalamic (CT) input to the dLGN has been shown to regulate tonic and burst firing modes [6], [7], contribute to the receptive field structure of dLGN neurons [8]–[10] and synchronize activity between the thalamus and cortex in a stimulus-specific manner [11], [12]. Nonetheless, the cellular mechanisms underlying these effects remain unresolved, in part because the CT pathway innervates a number of different thalamic cell types that are also connected to each other (Fig. 1). In addition to providing direct excitatory input to dLGN relay cells [5], [13], CT afferents send collaterals to the visual sector of the adjacent thalamic reticular nucleus (TRN) [14], [15], which contain GABAergic neurons that make feedback connections with dLGN cells [16]–[18]. CT inputs to the dLGN also innervate GABAergic intrinsic interneurons [4], [19], [20] that synapse directly onto relay cells [5], [21]. Thus the CT pathway, while providing direct excitation to these three cell types, can also activate convergent inhibitory input onto relay cells.

While the slice preparation has proven to be a valuable assay, the reliance on electrical stimulation to untangle the interactions between CT neurons and different thalamic cell types has been challenging, and in some cases has produced variable results [22]–[27]. Besides the obvious difficulty in preserving CT connections in a slice preparation, the placement of stimulating electrodes in adjacent thalamic nuclei to stimulate CT axons can lead to the inadvertent activation of other thalamic circuits, such as those between the dLGN and the TRN [28], [29] or even those that involve ascending cholinergic projections arising from the brainstem [30], [31]. These problems are further exacerbated when attempting to study the effects of CT stimulation in the developing thalamus, especially at postnatal ages when CT projections are beginning to innervate the dorsal thalamus [32]. To overcome these technical obstacles, and to gain further insight as to how CT input affects the activity of different thalamic cell types, we took an optogenetic approach.

By injecting an adeno-associated virus (AAV) containing a mammalian codon optimized chimera of channelrhodopsin 1 and 2 [33] fused to tdTomato (ChIEF-tdTomato) into the mouse visual cortex, we were able to express ChIEF-tdTomato in the somata, dendrites and axon terminals of layer VI neurons. Such expression allowed for the direct and selective photoactivation of CT terminal arbors in slice preparations that contained the dLGN or the TRN. Furthermore, by using transgenic mouse lines that express GFP under the control of promoters for different isoforms of glutamic acid decarboxylase (GAD), we were able to readily identify and target recordings to intrinsic interneurons in the dLGN (GAD67-GFP) [34] and GABAergic neurons in the visual sector of the TRN (GAD65-GFP) [35]. This approach allowed us to examine and compare CT-evoked synaptic responses in relay cells and interneurons in the dLGN as well as GABAergic neurons in the TRN. Moreover, we were able for the first time to examine CT responses in the developing dLGN at postnatal ages when CT axons have just finished innervating the dorsal thalamus [32].

## Methods

### Animals

All breeding and experimental procedures were approved by the Virginia Commonwealth University Institutional Animal Care and Use Committee (protocol AD20205). Experiments were carried out using three different transgenic strains in which GFP was used to visualize either layer VI neurons in the neocortex (golli-τ-GFP) [32], GABAergic neurons in the TRN (GAD65-GFP) or intrinsic interneurons in the dLGN (GAD67-GFP; JAX stock #007677). In the golli-τ-GFP mouse, the golli promoter drives the expression of a tau-enhanced green fluorescent protein (τ-GFP) fusion protein in layer VI neurons including their descending projections throughout thalamus. In the GAD65-GFP mouse, the GAD65 promoter drives the expression of GFP in GABAergic neurons in the TRN, but not the dLGN. In the GAD67-GFP mouse, the GAD67 promoter drives the expression of GFP in intrinsic GABAergic interneurons in the dLGN, but not the TRN. All founder lines were on a pigmented background (i.e., CB6F1/J, B6;129SV or C57/BL6). In total, experiments were performed on 57 mice (19 golli-τ-GFP, 15 GAD65-GFP and 23 GAD67-GFP).

### Virus Injections

To photoactivate CT terminal arbors in the dLGN or the TRN, an AAV carrying a vector for ChIEF fused to tdTomato was injected into layer VI of the visual cortex. To make this vector, cDNA from a mammalian codon optimized clone of ChIEF, fused to tdTomato, was cut out of pCAGGS-I-oChIEF-tdTomato-I-WPRE (gift of R. Tsien) and ligated into pACAGW-ChR2-Venus-AAV backbone (donated by K. Svoboda; Addgene #20071) at Age I and Xho I sites. This pAAV cis-plasmid was sent to Vector Biolabs for large-scale production of the AAV (AAV-ChIEF-tdTomato) (serotype 2/1, titer = 2.0×10^12 ^VG/ml).

For virus delivery, mice ranging in age between postnatal day (P) 0–16 were deeply anesthetized with isoflurane. An incision was made along the scalp, and a small hole created in the skull above the visual cortex. Virus (1–2 µl total volume) was delivered via a glass micropipette (placed in the deep layers of visual cortex) using air pressure applied from a Picospritzer II (General Valve Corporation, 5–10 psi, 2–5 msec duration, ≤1 pulse per sec). Typically two injections were made in each hemisphere and gave rise to widespread expression throughout layer VI of the visual cortex. Post-surgery, animals were carefully monitored for proper wound healing, assessed for signs of pain or distress and administered carprofen (5 mg/kg every 24 hours) and buprenorphine (0.1 mg/kg every 12 hours) for up to 3 days.

### Slice Preparation and in vitro Recording

Ten to 14 days following injection of the AAV, mice were deeply anesthetized with isoflurane and rapidly decapitated. The brain was removed, hemisectioned, and immersed in an ice cold oxygenated sucrose cutting solution containing (in mM): sucrose 230, KCl 2.5, CaCl_2_ 2, MgCl_2_ 6, NaHPO_4_ 1, NaHCO_3_ 25, glucose 25) and sectioned into 300 µm thick slices using a Leica VT1000S vibratome. For dLGN and visual cortex recordings, the brain was cut in the coronal plane. For TRN recordings, the brain was cut horizontally with the anterior aspect elevated at an angle of ∼20°. Tissue sections were incubated in a holding chamber of oxygenated ACSF, containing (in mM): NaCl 126, NaHCO_3_ 26, glucose 10, KCl 2.5, MgCl_2_ 2, CaCl_2_ 2, NaH_2_PO_4_ 1.25, at 35°C for 30 min and then brought to room temperature. Individual slices were then transferred to a recording chamber maintained at 32°C, and perfused continuously at a rate of 2–3 ml/min with oxygenated ACSF.

Neurons were visualized on an upright microscope (Olympus BX51WI) equipped with both DIC optics and filter sets for visualizing GFP (Chroma 49002) and tdTomato (Chroma 49005) using a 10X or 60X water immersion objective (Olympus USA) and a CCD camera. Patch electrodes were pulled vertically in two stages from borosilicate glass and filled with (in mM): KMeSO_4_ 135, NaCl 8, MgATP 2, NaGTP 0.3, HEPES 10, K_4_-BAPTA 0.1. The final tip resistance of filled electrodes was 3–6 MΩ. Whole-cell current and voltage clamp recordings were made using a Multiclamp 700B amplifier (Molecular Devices), filtered at 3–10 kHz, digitized at 10–100 kHz through an A/D board (National Instruments PCI-6221) and stored directly on a computer. Data acquisition and analysis was accomplished by using Strathclyde Electrophysiology Software (Whole Cell Analysis Program V3.8.2). Pipette capacitance, series resistance and whole cell capacitance were carefully monitored and compensated electronically during the recording. Voltage clamp recordings were performed without series resistance compensation. Recordings were made from cells with a resting membrane potential between −55 and −75 mV and a series resistance between 10 and 25 MΩ. Only experiments in which series resistance remained relatively stable (<25% change) were included for analysis. No significant differences in series resistance were found between different cell types (relay cells, 14.2±5.1; TRN neurons, 14.0±4.6; dLGN interneurons, 15.4±6.1 M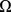; one-way ANOVA, F = 0.55, p>0.5). Membrane potentials have been corrected for the calculated error resulting from the liquid junction potential (13 mV). Unless otherwise noted, the holding potential was −70 mV.

For photoactivation, light from a 150W Xenon arc lamp was bandpass filtered (450–490 nm; EGFP filterset, HQ470/40X exciter, Chroma 41018), and reflected into a 10X or 60X objective. This produced a spot of blue light onto the submerged slice with an approximate diameter of 2.2 or 0.45 mm, respectively. In some experiments, the epifluorescence apertures were adjusted to produce a small spot of light through the 60X objective with an approximate diameter of 25 µm. Light was gated using a shutter (Uniblitz VS25, Vincent Associates) in which pulse duration and frequency were under computer control. For repetitive stimulation, pulse duration was between 2–7 msec.

For recordings designed to assess the underlying pharmacology of synaptic events, a number of different ligand or voltage-gated ion channel antagonists were bath applied. The GABA_A_ and GABA_B_ antagonists, bicuculline methochloride (50 µM, Ascent Scientific Asc110) and CGP 55845 (1 µM, Tocris 1248) were used to block inhibitory postsynaptic activity. The AMPA and NMDA antagonists, NBQX (10 µM, Ascent Scientific Acs046) and APV (50 µM, Ascent Scientific Asc003) were used to block excitatory events. The metabotropic glutamate receptor 1α (mGluR_1α_) antagonist, LY367385 (100 µM, Tocris 1237) and the mGluR_5_ antagonist, MTEP (100 µM, Tocris 2921) were used to block Group I mGluRs. Nimodipine (10 µM, Tocris 0600) was utilized to block L-type Ca^2+^ channels, while the voltage-gated Na^+^ channel blocker tetrodotoxin citrate (TTX) (1 µM, Tocris 1069) was used to abolish action potential-mediated synaptic transmitter release.

The amplitude of synaptic responses was measured from baseline values obtained just prior to photostimulation. Evoked responses <6 pA were below our detection limit (set at ∼2 times the average RMS noise) and thus excluded from the analysis. To quantify the degree of facilitation in responses evoked by repetitive trains of light, the amplitude of the *n* ^th^ response was divided by the amplitude of the initial response, and then multiplied by 100. Typically, amplitude values were based on the average of 3–7 stimulus presentations. Descriptive and inferential statistics were performed using Prism 5 (Graphpad) and pCLAMP 9 (Molecular Devices).

### Histology

In some recordings, Biocytin (0.1–0.2% w/v; Molecular Probes B1592) was included in the patch electrode and allowed to diffuse into the cell during recording. After the conclusion of recordings, the slice was placed in a fixative solution consisting of 4% paraformaldehyde in phosphate-buffered saline (PBS). Slices were kept in this solution overnight at 4°C, and then washed in PBS. For labeling, slices were treated with 0.1% Triton X-100 and Alexa Fluor 647 conjugated strepavadin (1∶1000, Invitrogen S21374) in PBS for 24 hours. Slices were then rinsed with PBS, mounted with Prolong Gold (Invitrogen P36930) and cover slipped. Epifluorescent images of fixed slices and filled cells were collected with a Nikon E600 upright microscope, and in some cases, confocal images were collected with a Zeiss LSM 510 Meta confocal microscope.

## Results

### Expression of ChIEF-tdTomato in CT Neurons

**Figure 2:**
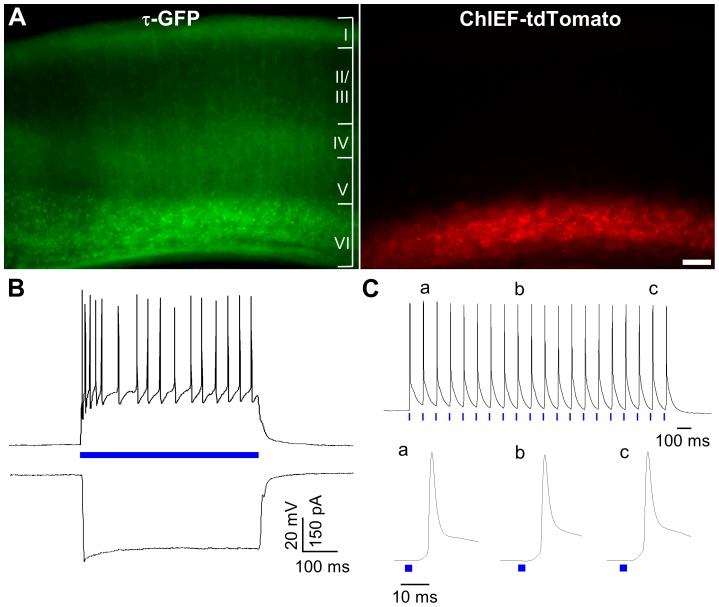
ChIEF-tdTomato expression in the visual cortex of the golli-τ-GFP mouse. A) Expression of τ-GFP (*left*) and ChIEF-tdTomato (*right*) in a 300 µm thick slice that was fixed overnight after *in vitro* recording. Note the correspondence of τ-GFP and tdTomato expression in layer VI. Because the tdTomato expression was so high in somata, labeled dendritic processes are obscured in the photomicrograph. Regions corresponding to different cortical layers are delineated in roman numerals. B) Current (*above*) and voltage clamp (*below*) responses of a layer VI neuron evoked by a single 500 ms pulse of blue light (blue bar) were large (∼30 mV, ∼400 pA) and closely matched stimulus duration. In current clamp (V_m_ = −70 mV), a train of action potentials rides the top of the sustained depolarization, while in voltage clamp (V_H_ = −70 mV) the light-evoked current persisted throughout the duration of the pulse showing little if any inactivation. Scale bar = 100 µm. C) Current clamp responses of a different neuron (V_m_ = −78 mV) to a small spot of blue light (25 µm diameter) that was directed at CT axons in the white matter about ∼500 µm from the patched cell. A 10 Hz (20 pulse) train of blue light generated a sequence of action potentials. Below the spike train are expanded traces of representative action potentials evoked by the 2^nd^ (a), 9^th^ (b) and 19^th^ (c) pulse. Each pulse generated a single, fast rising action potential. For B and C, responses were recorded in the presence of NBQX (10 µM), APV (10 µM), bicuculline (50 µM) and CGP 55845 (1 µM).

Targeted injections of the AAV construct containing the coding sequence for ChIEF-tdTomato into the deep layers of the visual cortex of golli-τ-GFP mice resulted in strong expression in layer VI neurons (Fig. 2A). Indeed, there was a high degree of overlap between τ-GFP labeled neurons (Fig. 2A, *left*), which are found throughout layer VI and project their axons into the thalamus [32], and regions that expressed ChIEF-tdTomato (Fig. 2A, *right*).

To confirm that we could reliably activate layer VI neurons with blue light, whole-cell recordings were made from visually identified pyramidal cells expressing ChIEF-tdTomato (n = 4). Representative recordings are shown in Fig. 2B–C. In these experiments, light-evoked activity was isolated by bath applying the glutamate receptor antagonists NBQX (10 µM) and APV (50 µM) as well as the GABA receptor antagonists bicuculline (50 µM) and CGP 55845 (1 µM). Long single pulses of blue light (500 ms) focused on the soma, resulted in a sustained depolarization (20–50 mV) and a train of action potentials that lasted throughout the period of light stimulation (Fig. 2B, *upper trace*). The depolarization exhibited relatively fast kinetics that corresponded to the onset and termination of the light pulse. Furthermore, corresponding voltage clamp recordings (Fig. 2B, *lower trace*) revealed that photocurrents were large (250–500 pA), had relatively fast kinetics and showed little inactivation. To ensure that action potentials could be initiated in axons, we presented small spots of blue light (∼25 µm diameter) within the white matter, ∼400–700 µm from the patched cell. As shown by the example in Fig. 2C, a train of pulses (10 Hz, 2 ms duration, 20 pulses) gave rise to repetitive spike firing. The expanded traces in Fig. 2C, reveal that each pulse evoked a single, fast rising action potential. Taken together these recordings demonstrate that light activation of ChIEF expressing neurons results in strong levels of depolarization that can lead to reliable action potential firing.

### dLGN Relay Cells

**Figure 3:**
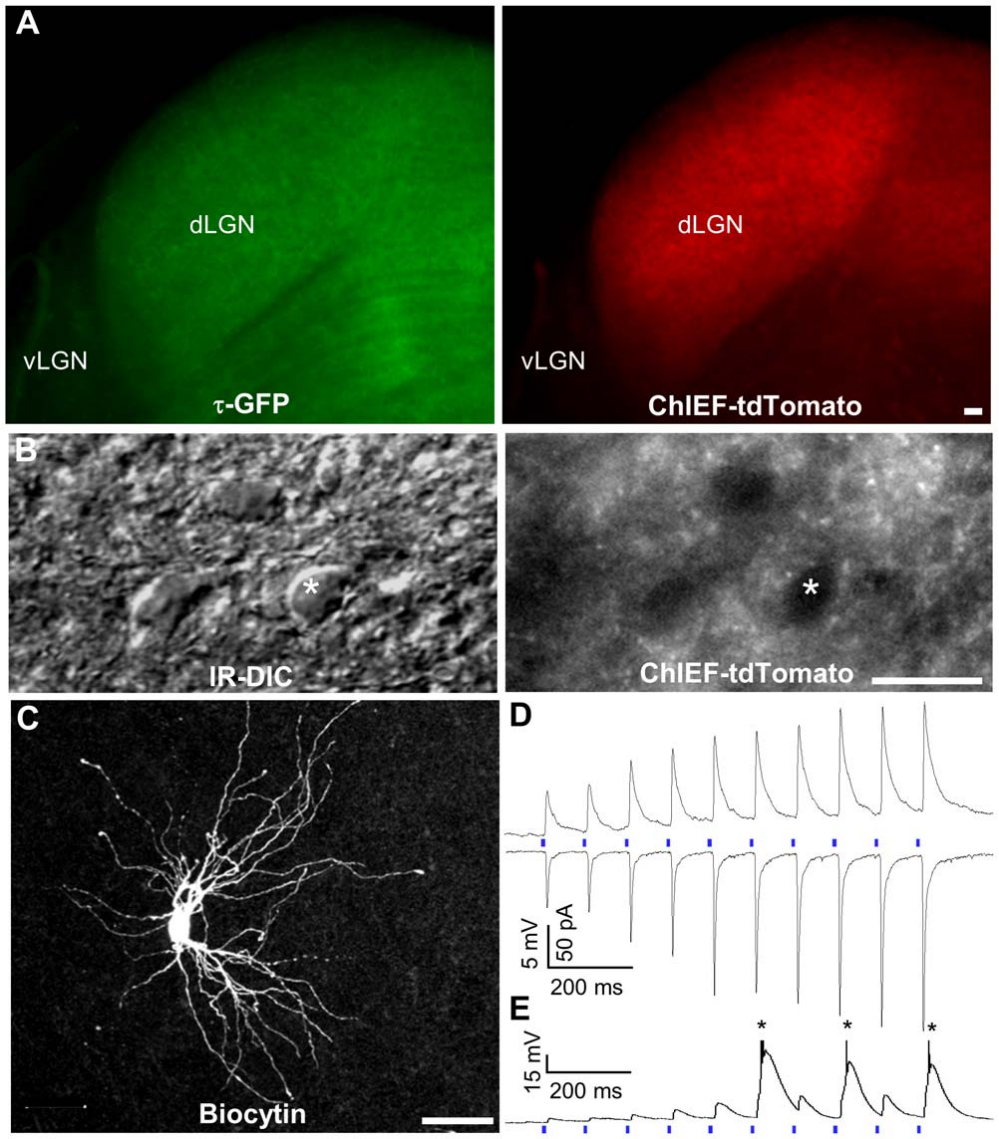
ChIEF-tdTomato expression and light-evoked synaptic responses of relay cells in the dLGN of the golli-τ-GFP mouse. A) Comparison of τ-GFP (*left*) and ChIEF-tdTomato (*right*) in a 300 µm thick slice containing dLGN that was fixed overnight after *in vitro* recording. Layer VI injections led to widespread expression throughout the dLGN that overlapped with τ-GFP labeled CT projections. vLGN, ventral lateal geniculate nucleus; dLGN, dorsal lateral geniculate nucleus. B) High magnification IR-DIC image (*left*) of the dLGN taken during *in vitro* recording and the corresponding fluorescence image (*right*) showing dense expression of tdTomato terminals surrounding the neuron targeted for whole cell recording (asterisk). C) Confocal image stack of the relay neuron recorded in B that was filled with biocytin during recording. Scale bar = 25 µm. D) Whole-cell current (*top*, V_m_ = −70 mV) and voltage (*bottom*, V_H_ = −70 mV) clamp recordings from the same neuron showing that repetitive photostimulation (10 Hz, blue bars) of ChIEF-expressing CT arbors produced a train of EPSPs and EPSCs that exhibited facilitation. E) Whole-cell current clamp recording from another neuron (V_m_ = −80 mV) illustrating that repetitive stimulation could evoke sufficiently large EPSPs to activate low-threshold (LT) Ca^2+^ spikes and burst responses (asterisks). Action potentials are truncated for clarity.

Cortical injections of AAV-ChIEF-tdTomato also gave rise to robust expression in CT axons innervating the dorsal thalamus. We routinely observed widespread expression of ChIEF-tdTomato that overlapped with τ-GFP throughout the dLGN (Fig. 3A). Importantly, no somatic labeling was observed, indicating that expression in the thalamus was restricted to CT axons and their terminal fields. As shown in DIC and fluorescence images from an *in vitro* slice in Fig. 3B, a dense plexus of labeled axons and terminals could be seen surrounding somata that were targeted for recording.

Whole-cell recordings were obtained from a total of 76 relay neurons between P10–30. Under DIC optics, relay cells could be distinguished from interneurons by their large round somata (Fig. 3B). Their identity was further verified by their electrophysiological properties and in many cases (n = 50) by their dendritic morphology, which was reconstructed from biocytin fills conducted during the recording. As shown in Figs. 3C and 5B, relay cells were readily distinguished from interneurons, having type I or class A morphology that consisted of a thick unbranched axon, large round somata, and multipolar dendritic arbors with as many as six to seven primary dendrites [36]–[39].

At late postnatal ages (P21–30), when dLGN cells are considered adult-like [38], [39] and well after the time CT axons innervate the dorsal thalamus [32], repetitive blue light stimulation evoked large and reliable excitatory postsynaptic potentials (EPSPs) and currents (EPSCs) that exhibited strong facilitation. Representative examples are shown in Fig. 3D–E, in which a 10 Hz train (10 pulses) evoked postsynaptic activity that increased in amplitude with each successive pulse. Moreover, at hyperpolarized potentials (e.g., ≤−70 mV), EPSPs often evoked low-threshold (LT) Ca^2+^ spikes and burst responses (n = 10).

**Figure 4:**
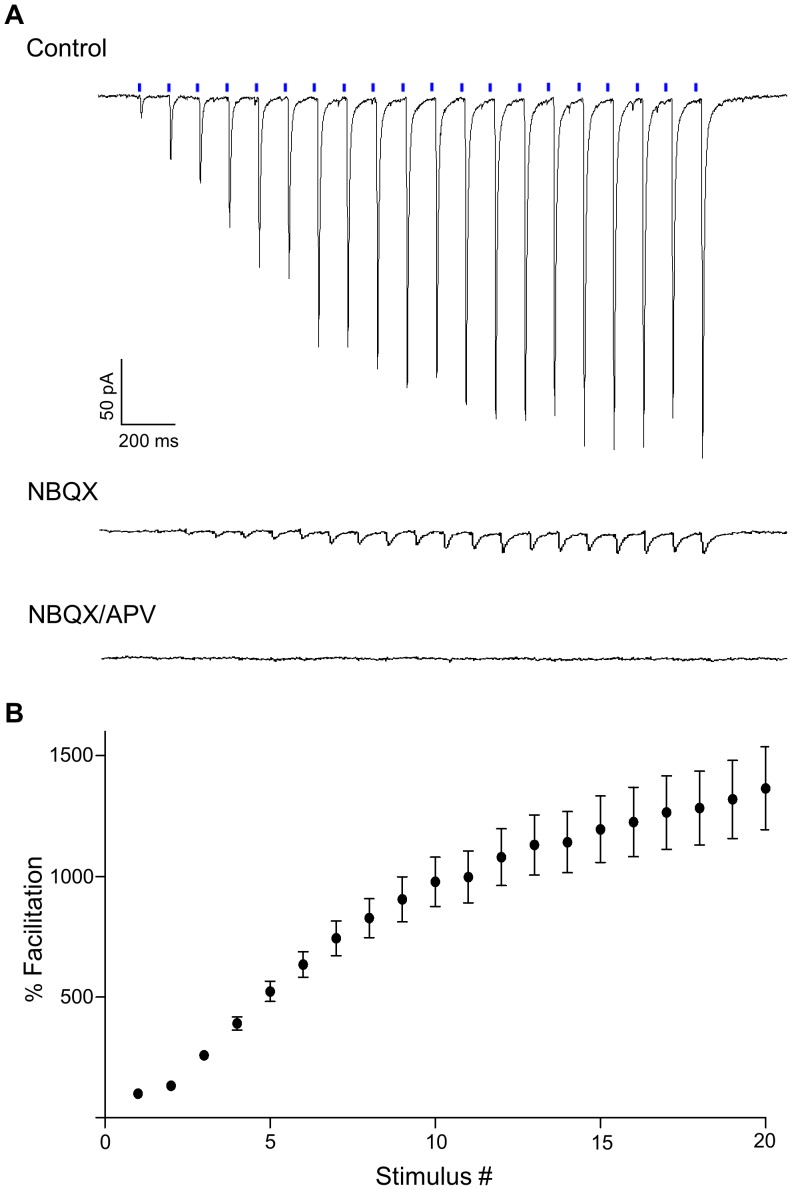
Facilitation and pharmacology of optically-evoked CT EPSCs in dLGN relay neurons. A) Example of EPSCs recorded from a relay neuron (V_H_ = −70 mV) evoked by a 10 Hz train of blue light pulses (20 pulses, blue bars). Bath application of NBQX (10 µM) greatly reduced the amplitude of evoked currents while subsequent application of APV (50 µM) abolished the remaining response. B) Summary plot showing the degree of facilitation of light-evoked CT EPSCs as a function of stimulus number for all relay neurons (n = 31). To calculate the percent facilitation, the amplitude of the *n* ^th^ response was divided by the amplitude of the initial response, and multiplied by 100. Each point represents the mean and bars indicate ±SEM.

To quantify the degree of facilitation evoked by repetitive CT stimulation, we used a 10 Hz, 20 pulse-train of blue light (Fig. 4A, *top*). Even responses to trains of this length continued to exhibit strong facilitation. Fig. 4B summarizes the responses of 31 relay cells by plotting the degree of facilitation as a function of stimulus number. Responses grew rapidly, but saturated by the 13^th^ pulse (repeated measures ANOVA, F = 48.87; Dunnett’s *post hoc* test; pulses 1–12 *vs* 20, all p values <0.05; pulses 13–19 *vs* pulse 20, all p values >0.05), reaching a peak amplitude that was 1351±171.1% of the initial EPSC. To confirm these light-evoked EPSCs were due to the release of glutamate at CT terminals, we bath applied the AMPA receptor antagonist NBQX (10 µM), which in some cases was followed by the NMDA receptor blocker APV (50 µM). An example of light-evoked responses recorded before and after antagonist applications is shown in Fig. 4A. Overall, NBQX greatly reduced the response across each successive pulse (e.g., 91.8±1.4% at the 18^th^ pulse, n = 9), while the subsequent application of APV nearly abolished the remaining response (99.5±0.2% of control, n = 6).

**Figure 5:**
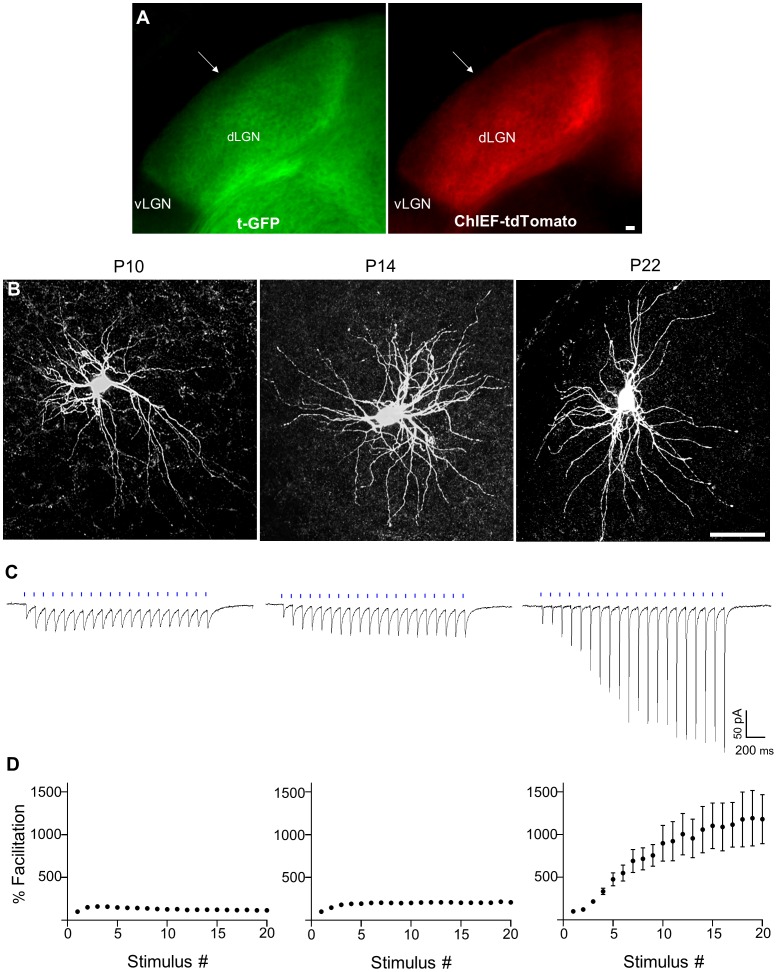
ChIEF-tdTomato expression and light-evoked responses in relay cells of the dLGN at different postnatal (P) ages. A) Comparison of τ-GFP (*left*) and ChIEF-tdTomato (*right*) in a slice containing dLGN at P10. Arrows highlight a dorsolateral region just beneath the optic tract that lacks label, indicating that CT arbors have not yet innervated this area. Even at this age, cortical injections gave rise to a high level of ChIEF-tdTomato expression that matched the pattern of GFP-labeling. B) Z-stack projection images of relay cells at P10, P14 and P22 that were filled with biocytin during recording. Scale bar = 25 µm. C) CT-evoked responses from individual relay neurons to a 10 Hz train (20 pulses, blue bars) at the same ages shown in B. (D) Below each response is the corresponding summary plot showing the degree of facilitation (mean±SEM) as a function of stimulus number for the total number of neurons recorded at each age (P10, n = 22; P14, n = 16; P22 n = 7). For P10 and P14 error bars are too small to be seen. For P22, additional recordings were made from those summarized in Fig 3.

To study the development of CT responses in relay cells, cortical injections of AAV-ChIEF-tdTomato were made at early postnatal ages (P0, 4 and 12) and *in vitro* recordings were then conducted at P10 (n = 22), P14 (n = 16), and P22 (n = 7). Such timing corresponded to ages when CT projections begin to innervate the dorsal thalamus (P4–7) [32] and form synapses with dLGN relay cells (P7–14) [5]. As shown in Fig. 5A, by utilizing the golli-τ-GFP mouse we could verify that at the earliest age of recording (P10), CT arbors innervate nearly the entire dLGN and express high levels ChIEF-tdTomato. Representative examples of biocytin filled relay cells and corresponding CT-evoked responses are shown in Fig. 5B–C. Light-evoked responses were evident as early as P10 and the initial response to the stimulus train (10 Hz, 20 pulses) was similar in amplitude to those recorded at P14 and P22 (P10, 25.9±5 pA; P14, 28.9±10 pA; P22, 27.9±9.3 pA; one-way ANOVA, F = 0.1, p>0.9). However, maximal amplitude increased with age (P10, 24.8±3.7 pA; P14, 75.5±15 pA; P22, 288±103.1 pA; one-way ANOVA, F = 15.77; tukey’s *post hoc* test; P10 *vs* P14, p>0.4; P10 *vs* P22, p<0.0001; P14 *vs* P22, p<0.0001) and occurred at different stimulus intervals (Fig. 5D). For example, for both P10 and P14, the maximal amplitude occurred as early as the 2^nd^ or 3^rd^ pulse, whereas at P22, peak values were evident near the end of the stimulus train (18–20^th^ pulse, see also Fig. 4B). Moreover, as shown in the summary plots of Fig. 5D, at P10 and P14 facilitation was transient and relatively weak. For example, at P10 facilitation peaked by the 3^rd^ stimulus pulse (160.3±8.5%) and returned to near baseline values by the end of the stimulus train (117.1±7.6%, Fig. 5D, *left*). Similarly, at P14 facilitation was still weak, peaking around the 4^th^ pulse (204.6±17.8%, Fig. 5D, *middle*), but sustaining these levels throughout the remainder of the train (20^th^ pulse, 209.0±22.0%). As noted earlier, the degree of facilitation at late ages (>P21) was far greater (see Fig. 4B). For comparison, at P22 facilitation grew rapidly and persisted throughout the train, reaching a value of 1192.0±324.1% (n = 7) of the initial amplitude (Fig. 5D, *right*).

### TRN Neurons

**Figure 6:**
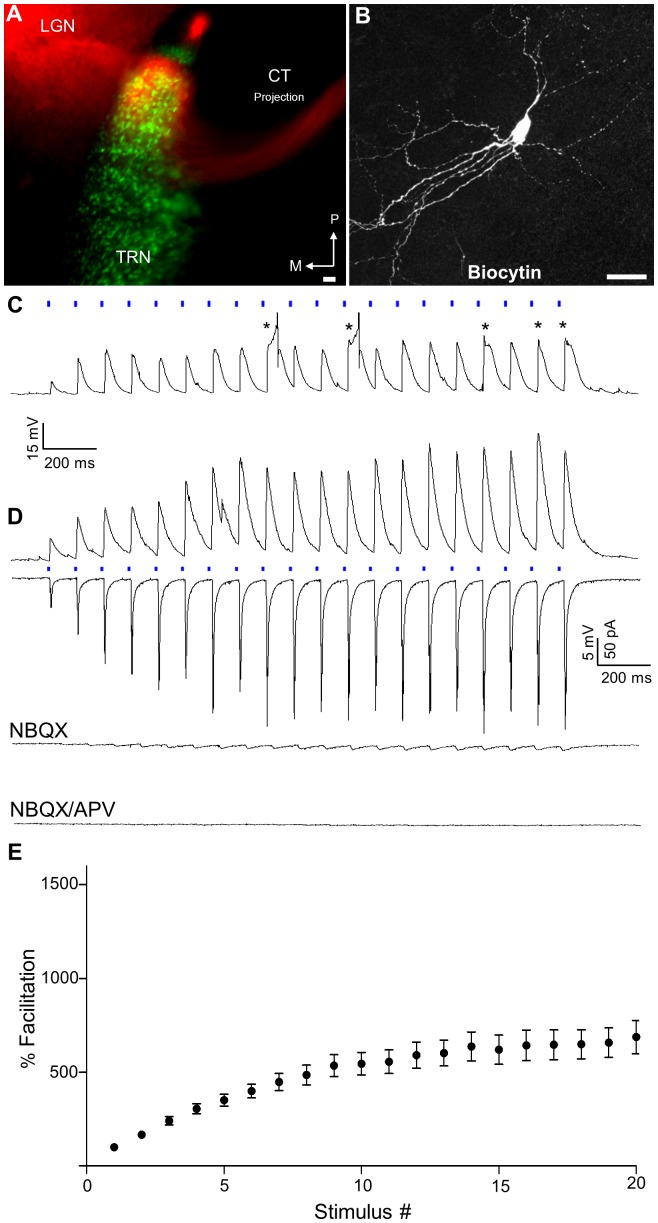
ChIEF-tdTomato expression of CT projections and light-evoked synaptic responses in GABAergic TRN neurons of the GAD65-GFP mouse. A) Superimposed fluorescence images of a 300 µm thick horizontal slice from a GAD65-GFP mouse that was fixed overnight following *in vitro* recordings. CT projections expressing ChIEF-tdTomato (red) can be seen in regions that contain GAD65-GFP positive TRN neurons (green). Areas in yellow depict overlap. P, posterior; M, medial. B) Confocal image stack of a TRN neuron filled with biocytin during recording. Scale bars = 25 µm. C) Example of a whole-cell current clamp recording (V_m_ = −85 mV) showing that repetitive stimulation led to EPSPs that could evoke LT Ca^2+^ spikes and burst responses (asterisks). Action potentials are truncated for clarity. D) Example of EPSPs (V_m_ = −70 mV) and corresponding EPSCs (V_H_ = −70 mV) recorded from another GFP positive TRN neuron. Responses were evoked by a 10 Hz train of blue light (20 pulses, blue bars) and showed strong facilitation. Bath application of NBQX (10 µM) greatly reduced the amplitude of evoked currents while subsequent application of APV (50 µM) abolished the remaining response. E) Summary plot showing the degree of facilitation (mean±SEM) as a function of stimulus number for all TRN neurons (n = 31).

To examine CT responses in GABAergic TRN neurons, we made cortical injections of AAV-ChIEF-tdTomato in mature (P20–30) GAD65-GFP mice. Using a horizontal slice for recording, we readily observed strong expression ChIEF-tdTomato in CT axons and terminal fields that overlapped with GAD65-GFP neurons residing in the visual sector of the TRN (Fig. 6A). Targeted recordings with biocytin filled electrodes revealed that these GFP positive cells (n = 15) had fusiform-shaped somata that were confined to the boundaries of TRN, and either bipolar or multipolar primary dendrites (Fig. 6B).

Similar to the dLGN relay cells, TRN neurons also exhibited strong facilitating responses to repetitive stimulation (Fig. 6C–D). Additionally, at hyperpolarized membrane potentials EPSPs were capable of eliciting LT Ca^2+^ spikes and burst responses (n = 8; Fig. 6C). The summary plot in Fig. 6E for TRN neurons shows that responses grew quickly, began to plateau by the 12^th^ pulse (repeated measures ANOVA, F = 34.31; Dunnett’s *post hoc* test; pulses 1–11 *vs* 20, all p values <0.05; pulses 12–19 *vs* 20, all p values p>0.05), and peaked at a value that was 721.8±88.9% (n = 24) of the initial EPSC. Bath application of NBQX (10 µM), and then APV (50 µM) revealed that responses were mediated almost entirely by AMPA and NMDA receptor activation (Fig. 6D). NBQX reduced the response by 91.3±4.8% (n = 10) compared to control, while the addition of APV all but abolished the residual response (99.7±0.2% of control, n = 5).

### Comparison of dLGN Relay Cells and TRN Neurons

**Figure 7:**
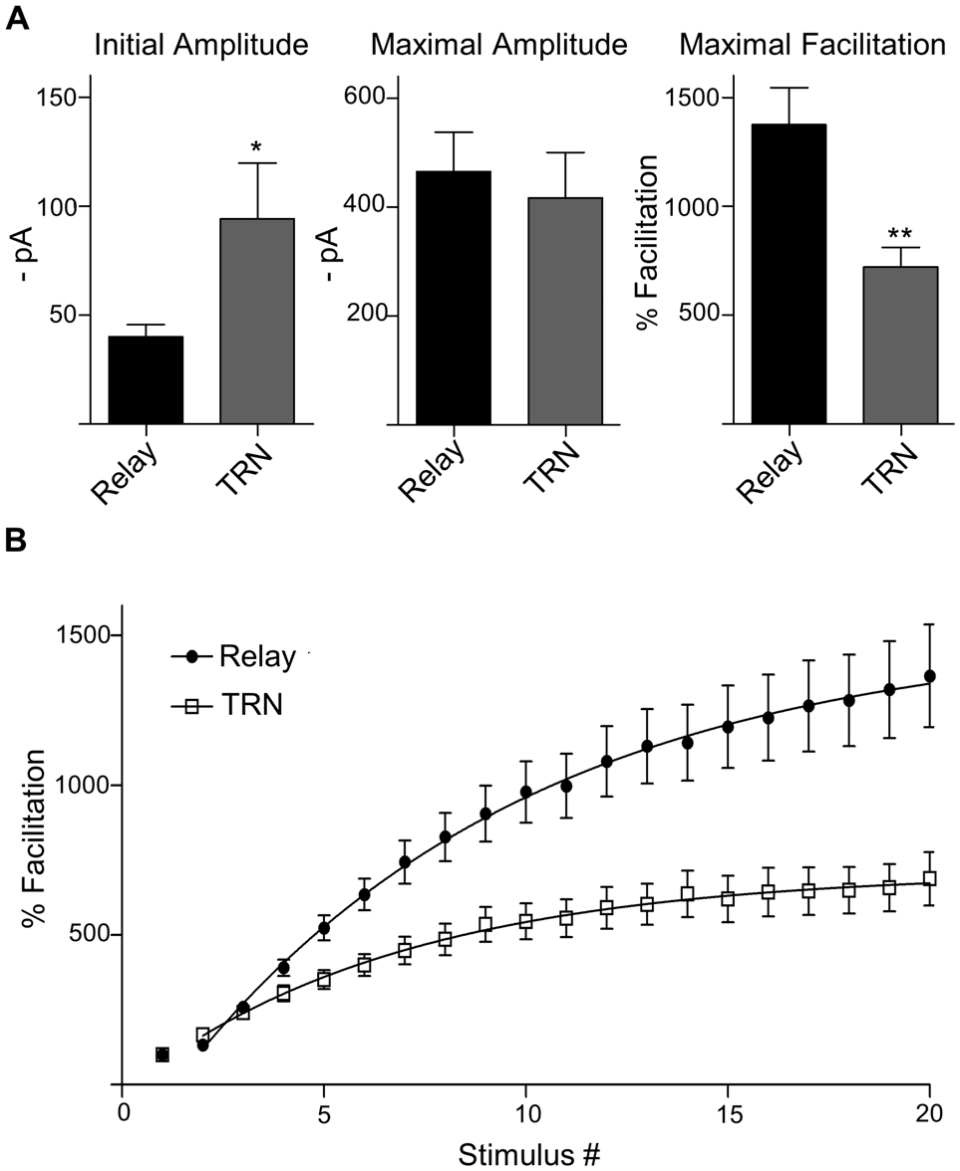
Comparison of optically-evoked CT responses in relay cells and GABAergic TRN neurons. A) Summary plots showing the means (±SEM) for initial amplitude, maximal amplitude and maximum facilitation for relay cells and TRN neurons. Initial amplitude of relay cells was smaller, but had comparable maximal values thus exhibiting a greater degree of facilitation than TRN neurons (*p<0.05; **p<0.01). B) Plots for mean % facilitation (±SEM) as a function of stimulus number for relay and TRN neurons. Starting with the 2^nd^ pulse, plots were best fit with a single exponential. A comparison of tau (relay, 10.1; TRN, 7.1) revealed that facilitation grew faster and plateaued for TRN neurons.

While CT evoked EPSCs facilitate in both relay and TRN neurons, differences in the degree and kinetics have been reported [24], [40]. To examine this in our sample of optically-evoked responses, we compared the initial and maximal amplitudes, maximal facilitation, and the rate of facilitation as a function of stimulus number (Fig. 7A–B). We found the amplitude of the initial response was significantly greater for TRN compared to dLGN relay neurons (TRN, 94.2±25.6 pA; relay, 40.1±5.5 pA; two tailed t-test, p<0.05). However, maximal amplitudes (20^th^ pulse) were not different from each other (TRN, 417.3±83.5 pA; relay, 465.5±72.4 pA; two tailed t-test, p>0.6). Thus, relay neurons exhibited about a 2-fold difference in the degree of facilitation compared to TRN neurons (relay, 1375.0±171.1%; TRN, 721.8±88.9%; Fishers exact test, p<0.01). To compare rates of facilitation, the degree of facilitation by stimulus number plots were best-fit by a single exponential function (Fig. 7B). Indeed, response amplitudes grew faster for TRN cells (tau = 7.1) when compared to relay neurons (tau = 10.1).

### dLGN Intrinsic Interneurons

**Figure 8:**
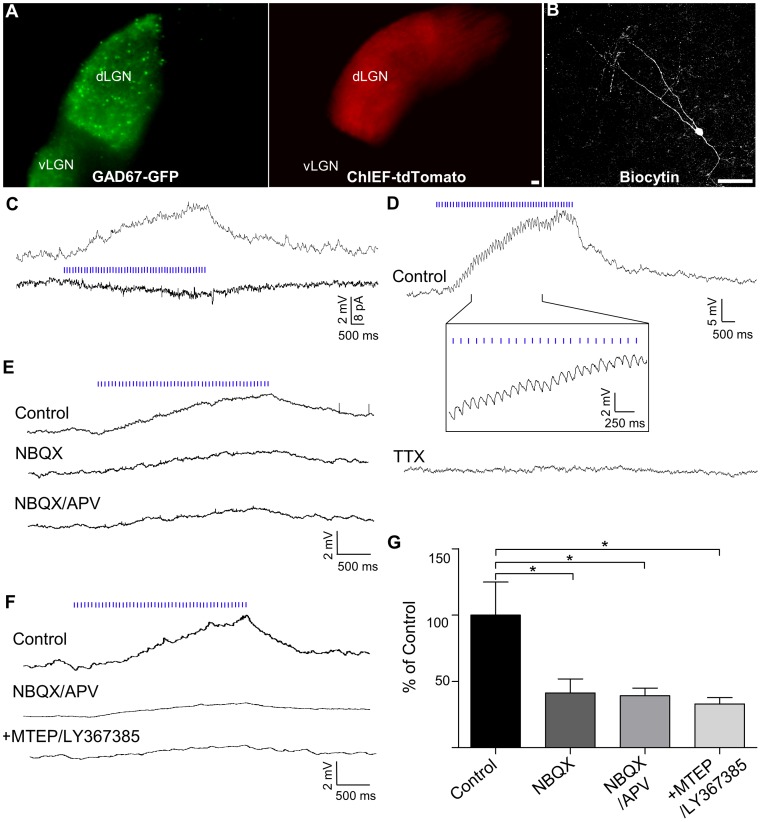
ChIEF-tdTomato expression of CT projections in dLGN and light-evoked synaptic responses of interneurons in the GAD67-GFP mouse. A) Example of a coronal slice used for recording showing GAD67-GFP positive interneurons (*left*) and widespread expression ChIEF-tdTomato expression (*right*) throughout dLGN. B) Confocal image stack of an interneuron filled with biocytin during recording. Scale bars = 25 µm. C) Whole-cell current (*top trace,* V_m_ = −70 mV) and voltage (*bottom trace,* V_H_ = −70 mV) clamp recordings from an LGN interneuron showing that repetitive photostimulation of CT fibers (10 Hz, 50 pulses; blue bars), produced a slow rising depolarization and underlying inward current that peaked near the termination of stimulation. D) Graded depolarization (*top trace*) recorded from an interneuron (V_m_ = −70 mV) evoked by a 10 Hz train of blue light pulses (50 pulses, blue bars). Expanded trace (*below*) illustrates the small unitary EPSPs that ride the crest of the slower depolarization. Bath application of TTX (1 µM), abolished both the graded depolarization and unitary EPSPs (*bottom trace*). E) Example of another response evoked by repetitive stimulation (20 Hz, 50 pulses, *top trace*). Bath application of NBQX (10 µM) significantly reduced the amplitude of depolarization (*middle trace*). The addition of APV (50 µM) had a negligible effect on the remaining depolarization (*bottom trace*). F) Excitatory response evoked by 20 Hz repetitive stimulation (50 pulses, *top trace*, V_m_ = −70 mV). Although bath application of NBQX (10 µM) and APV (50 µM) greatly reduced the response (*middle trace*), the addition of the Group I mGluR antagonists, MTEP and LY367381 had a no effect on the remaining depolarization (*bottom trace*). G) Summary plot of pharmacology experiments showing the mean reduction (±SEM) in the magnitude of the graded depolarization (pA x ms) expressed as a percentage of the control response. Application of NBQX reduced the response (n = 6) whereas the addition of APV (n = 7), or the Group I mGluR antagonists MTEP and LY367385 (n = 3) did not have any further effect on the remaining depolarization. (*p<0.05).

To examine CT responses among interneurons within the dLGN, we made cortical injections of AAV-ChIEF-tdTomato in GAD67-GFP mice. In coronal slices, GFP positive interneurons were found throughout the dLGN and were surrounded by a dense plexus of tdTomato labeled CT arbors (Fig. 8A). Recordings with biocytin filled electrodes showed that GFP positive neurons (n = 35) had morphology resembling intrinsic interneurons (Fig. 8B), having type II or class B features that consisted of a bipolar appearance with long thin dendrites emanating from the opposite poles of small, fusiform somata [36], [37], [41]. These morphological features give rise to electrotonic isolation of dendrites, and as a result introduce potential space clamp problems during somatic recordings [21], [42]–[44]. Since interneuron responses to CT stimulation obtained in the voltage clamp configuration were small and difficult to quantify (n = 5; Fig. 8C), we chose to conduct recordings in current clamp mode, which has the advantage of being less sensitive to changes in access resistance and more likely permitting for the detection of small synaptic responses. Whole-cell current clamp recordings were obtained from 83 interneurons from mice that were between P20–30. Despite the widespread expression of ChIEF-tdTomato throughout the dLGN, light-evoked responses to stimulus trains comprised of 20 or 50 pulses presented at 10 Hz or 20 Hz were relatively weak and somewhat variable in size with peak amplitudes ranging from 2–15 mV. In fact, for many cells (n = 46) it was difficult to detect light-evoked events. It is unlikely that such failures were due to the degree of ChIEF-tdTomato expression in dLGN, since large responses could be routinely evoked in relay neurons recorded within the same slice (n = 8, data not shown). For those cells that responded to optical stimulation (n = 37), repetitive pulses produced a slow rising, graded depolarization. In many instances (n = 20), a series of small, unitary, fast EPSPs that corresponded to the temporal rate of stimulation could be discerned riding the crest of the prolonged depolarization (Fig. 8D, inset).

To confirm these responses were synaptic in origin and dependent upon light-evoked action potentials generated in CT axons, we bath applied TTX (1 µM, n = 3). As illustrated in Fig. 8D, TTX blocked both the fast EPSPs as well as the graded depolarization. Finally, to assess the underlying pharmacology of these events, we bath applied the glutamate antagonists NBQX (10 µM) and APV (50 µM). As shown in Fig. 8E and G, NBQX reduced the postsynaptic response to a value that was 37.2% of control. However, the subsequent application of APV had little if any effect on the remaining response (one-way ANOVA, F = 29.45, tukey’s *post hoc* test, control *vs* NBQX, p<0.05, n = 6; control *vs* NBQX/APV, p<0.05, n = 7; NBQX vs NBQX/APV, p>0.05, n = 3). To assess whether the remaining response could be due to activation of Group I metabotropic glutamate receptors (mGluRs) [25], [45] we co-applied the mGluR_1α_ antagonist, LY367385 (100 µM) along with the mGluR_5_ antagonist, MTEP (100 µM). However, as illustrated in Fig.8F–G, the NBQX/APV-resistant component of the depolarization persisted in the presence of these metabotropic antagonists (one-way ANOVA, F = 29.45, tukey’s *post hoc* test p>0.05, n = 3). During one recording we applied the L-type Ca^2+^ channel antagonist, Nimodipine (10 µM) which also failed to affect the remaining response, suggesting that the depolarization recorded at the soma was not mediated by Group I mGluRs nor voltage-gated L-type Ca^2+^ channels [46], [47].

## Discussion

Our results reveal that the chimera of channelrhodopsins 1 and 2, known as ChIEF, is a highly effective optogenetic tool that can be used to study synaptic responses in slice preparations where it is difficult to isolate or preserve long range neuronal connections. AAV delivery of ChIEF produced strong and widespread expression in layer VI neurons of the visual cortex, which included their descending axons and terminal arbors throughout the visual sector of the thalamus. Blue light stimulation of their somata or distal axon segments, produced large currents and spike firing that was well correlated with the duration and pattern of light delivery. In thalamic slices where CT axons have been severed from their cell bodies, activation of their terminal arbors in dLGN and TRN also gave rise to robust excitatory postsynaptic activity, indicating that ChIEF can be used as a viable alternative to electrical stimulation. In fact, the light-gated properties of ChIEF channels offer some key advantages over other more conventional forms of channelrhodopsins. Most notable are the fast open and close times, and lack of desensitization during prolonged photostimulation [33], [48]. Such properties are especially useful for studies that involve repetitive stimulation, where a loss of current can lead to an artificial depression or blunting of responses [40], [49].

The use of ChIEF also allowed us to explore the ontogeny of CT responses in thalamus. As a first step we focused on dLGN relay cells, since our recent EM analysis of synaptic profiles in the dLGN suggests that synaptic connections between CT terminals and relay cells first appear sometime between first and second postnatal week [5]. Indeed, large excitatory responses were observed as early as P10 but the degree of facilitation induced by repetitive stimulation was relatively weak and transient, and did not appear fully mature until sometime between P14 and P21. Taken together, these results suggest that the age related changes in facilitation likely reflect the maturation of processes that regulate the synaptic vesicle cycle [50], [51]. One possibility is that synaptic proteins that aid in the recruitment of vesicles to active zones during high rates of stimulation have yet to mature. Most notable may be the synapsins, which in the dLGN have been found in corticothalamic terminals, and when genetically deleted lead to a decrease in short term facilitation of CT responses [26].

Our major findings with ChIEF showed that responses to CT stimulation varied among different classes of thalamic neurons. For relay cells of the dLGN and GABAergic cells of the TRN, repetitive stimulation produced a train of EPSCs that grew in amplitude with each successive pulse. These responses were mediated by ionotropic glutamatergic receptor activation and blocked by the bath application of NBQX and APV. While others have documented the involvement of metabotropic glutamate receptors [6], [22], [25], [52], their activation was not evident under our recording conditions. Indeed, such activation in the dLGN requires high rates of stimulation (e.g., 50–100 Hz) [6], [22], [25]. Unfortunately, our attempt to use such frequencies during photostimulation did not yield consistent or sufficient levels of depolarization, thereby compromising our ability to conduct a thorough examination of the underlying pharmacology. Such failure seems related to the temporal fidelity of CHIEF, while superior to other opsins is still limited, especially when high rates of stimulation are employed [33], [48], [49].

While facilitation of CT responses has been well documented in relay cells and TRN neurons [22], [24], [26], [27], [52]–[57], we noted differences in the initial amplitude, rate of growth, and degree of facilitation between relay and TRN neurons. For relay cells, the initial response was smaller, grew slower but had maximal amplitude that was similar to TRN cells. As a result, the degree of facilitation was about 2-fold larger for relay cells than TRN neurons, a pattern that is consistent with the CT responses reported in ferret dLGN and TRN [24], and mouse VB and TRN [40]. These differences in the initial amplitude and degree of facilitation are in part due to their postsynaptic AMPA GluR subunit composition. CT-TRN synapses possess a higher density of GluR4 subunits [15] thereby providing for stronger AMPA mediated responses that are likely to desensitize more quickly during repetitive stimulation [24], [58]–[61].

Thus, with a wider dynamic range, relay cells seem better suited to integrate the duration and intensity of CT input, a feature that has been implicated in synchronizing network activity between neocortex and thalamus during different behavioral states [60], [62]–[64] or sensory processing [11], [12]. Because of the inhibitory feedback connections between the TRN and the dLGN, CT activation of TRN neurons could further extend the dynamic range of relay cells, by dampening the initial excitation and spike generation of relay cells, thereby allowing them to show graded levels of depolarization during repeated stimulation. This balance in CT excitation and TRN feedback inhibition also seems critical for generation and maintenance of normal thalamocortical network oscillations. Indeed, weakening the excitatory strength of CT-TRN synapses results in increased CT relay cell excitability and the induction of abnormal paroxysmal activity [60].

In contrast to dLGN relay and TRN neurons, intrinsic interneurons in the dLGN responded to repetitive stimulation with a small, slow, graded depolarization, and in some instances, small unitary EPSPs rode the crest of this depolarization. Furthermore, a substantial component of synaptic responses recorded in interneurons remained after ionotropic glutamate receptor blockade. Pharmacological experiments suggested this residual response was not mediated by mGluRs [25] or the back propagation of voltage-gated L-type Ca^2+^ channels [46]. Although the underlying pharmacology of this response has yet to be determined, we cannot rule out the possibility that we failed to completely eliminate all postsynaptic glutamatergic transmission or that the remaining response reflected a photoelectrochemical artifact associated with blue light stimulation [65]–[67]. However, the latter seems unlikely since the residual depolarization persisted even when attempts were made to shield the recording pipette and ground wire.

The excitatory responses we recorded in interneurons after CT stimulation are similar to those reported in rat [25] but at odds with a recent report in the mouse dLGN, showing that paired or repetitive delivery of electrical shocks led to pronounced depression of excitatory responses [27]. Perhaps this discrepancy is related to the method of stimulation since electrical stimulation could potentially lead to inadvertent activation of interneuron circuits involving TRN and/or intranuclear axon collaterals of relay cells [13], [68], [69]. Nonetheless, the CT responses of interneurons evoked by photoactivation were much weaker than those recorded in dLGN relay and TRN neurons. In part, this could be due to the density and distribution of CT inputs onto these cell types. For example, interneurons receive far fewer CT inputs and these synapses appear to be located distally on the dendrites [4], [19], [20], [70]–[71]. Because interneurons have very long, small-diameter dendrites, distal synaptic events are subject to electrotonic isolation [21], [42]–[44]. Thus, the passive propagation of synaptic responses at these distant sites could lead to substantial attenuation and low-pass filtering of signals recorded at the soma. Nonetheless, it is clear that CT feedback leads to the excitation of intrinsic interneurons. Because of the inhibitory connections between interneurons and relay cells, such excitation would, as it does with the TRN, counteract the rapid initial CT induced depolarization of relay cells. Since feed-forward inhibition from interneurons onto relay cells contributes to their receptive field tuning [72]–[74], it is also conceivable that the improved stimulus specific tuning associated with CT activation could arise from the concerted CT activation of relay cells and interneurons [2], [11], [12], [75]. Finally it is worth noting that interneurons can inhibit relay cells through specialized F2 terminals, which form dendrodendritic synapses [5], [13], [21], [70], [76]. Since these F2 terminals are prevalent on distal dendritic sites, such an arrangement allows for highly localized interactions that can occur independent of somatic interneuron activity [43], [44], [46], [77].

In conclusion, these results reveal that optogenetics can be used effectively to examine long-range synaptic interactions in developing and mature circuits. When applied to the CT pathway they reveal that relay cells of dLGN neurons have a different excitatory profile than GABAergic TRN neurons and intrinsic interneurons. The concerted activation of GABAergic neurons during CT activation can further shape the excitatory responses of relay cells, extending their dynamic range during repetitive and sustained periods of stimulation. Understanding these differences in the pattern of excitation for different thalamic cell types should provide additional insight about how CT feedback alters the activity of visual thalamic circuitry during different behavioral or pathophysiological states.
